# Book Reviews

**Published:** 1806-11

**Authors:** 


### CRITICAL ANALYSIS

OF THE

### RECENT PUBLICATIONS

ON THE

### DIFFERENT BRANCHES OF PHYSIC, SURGERY, AND MEDICAL PHILOSOPHY.

---

### *The Edinburgh Medical and Surgical Journal,* Number 7.

Article 1.—*Account of the Illness and Death of* H. B. De Saussure, late Professor of Physic at Glasgow; *communicated by* Louis Odier, M. D. Professor of Medicine.

This is a very interesting communication. When medical men, high in the profession, have organic lesions, we have the fairest opportunities of learning all the symptoms during life, and comparing them with the probable causes by examination after death. The case, though related with great minuteness, is not too prolix. We are not perfectly satisfied with Dr. Odier's inductions, but as he states his facts and reasoning, we shall not interfere with a paper which can only be considered as a record, and consulted as such.

Article 2.—*Remarks on the White Induration of Organs.* By G. L. Bayle, M. D. Asistant Anatomist to the School of Medicine at Paris.

This paper is extracted from the Journal de Medicine. It is of a piece with many others we meet with from the same quarter. It seems to us to have been suggested by Mr. Abernethy's attempt at the classification of tumours. If we were dissatisfied with that gentleman's performance, we were at least ready to give credit for the goodness of his intentions, and to make large allowances for the novelty and boldness of the attempt. In the present instance we can hardly do so much. The whole, if it has any other meaning than an ostentatious display of something not understood by either the writer or reader, is much too deep for our comprehension. We hope, therefore, if our northern brethren should hereafter wish to borrow from such sources, that it will be only in those cases in which they can offer a few illustrations of their own.

Article 3.—*Observations on Tubercles found in the Brain of two scrofulous Subjects.* By F. V. Merat, M. D. Clinical Assistant at the School of Medicine at Paris.

Another paper from the same source. It is, however, less exceptionable than the former; and, as a record, might be worth preserving but not transcribing.

Article 4.—*History of a Case of diseased Spleen, with Appearances on Dissection.* By NATHAN DRAKE, M. D. Hadleigh, Suffolk.

OF this case we shall transcribe only the dissection, with a short remark of our own.

"*Appearances on Dissection.*

1. "The coats of the stomach were nearly three times thicker than usual; its cavity reduced to about one third of the customary size, the rugæ very distinct; the cardia and pylorus in a sound state; but a little above the sphincter pylori there was a quantity, nearly an ounce, of cheese-like matter in a granulated form, adhering to the external coat of the stomach, but not constricting the passage into the duodenum; there were no traces of inflammation either on the inner or outer coat of the stomach.

2. "The omentum was greatly enlarged, and entirely covered with cheese-like matter in a granulated state; considerable portions of its substance were thickened to a full inch in depth, and were altogether formed of the same caseous material, which might be rubbed between the fingers into a paste, but somewhat gritty to the touch. The omentum was depressed far below its usual situation, and strongly and extensively adhered to the peritonæum and intestines.

3. "The spleen was one mass of disease; half of its proper bulk appeared to be absorbed or wasted, and the organization of the remaining part completely obliterated, and in a state approaching towards solution; the peritonæal coat of its internal concave surface, was dilated into a very large cyst, with blood vessels of an enormous size, ramifying on its inferior part, or fundus. The upper part of the cyst strongly adhered to the whole under surface of the stomach, and the lower part to the upper edge of the great arch of the colon. The diameter of the cyst was full six inches, and it contained more than a pound and a half of dark, dense, coagulated blood, several portions of which, nearly as large as a man's fist, floated in a brown coloured serum, of which there was better than a pint. We have already related that two pints of a similar fluid had been drawn off, previous to death, and the capacity of the cyst was such as to admit of considerably more than four pints. The bottom and sides of this bag were covered about an inch deep, with a black tenacious matter of the consistence of congealed honey, and, when examined with the fingers, was found interspersed with masses of the same caseous substance, which covered the omentum. The great size of the cyst pressed the stomach high up, and close to the diaphragm, and the arch of the colon was, from the same cause, thrust down many inches below its natural situation, and was contracted in that part of its course to the size of a small intestine. There was no communication from this cyst with the liver, stomach, or intestines, and there was no fœtor, and no pus in any of the diseased viscera.

4. "The liver was pressed high up by the bulk of the cyst, its lower edge being considerably within the margin of the thorax. It was perfectly sound and healthy in all its lobes, not a mark of disease appearing, either externally or internally. It was rather smaller than usual in size, and the left lobe could not be said to pass into any part of the left hypochondriac region, owing probably to the magnitude of the cyst. The lobulus spigelii was also in the most perfect state, and the gall bladder was distended with bile, and of its proper colour.

5. "The intestines, with the exception of the arch of the colon, were nearly free from disease, but compressed into a very small compass, and adhering to the omentum. The blood vessels upon the arch of the colon were turgid, and part of the mesocolon was as much diseased as the omentum, being thickly interspersed with cheese-like matter in a granulated form. The arch of the colon was so reduced in bulk, and compressed, that when the body was opened, it projected like a chain of very small bladders.

6. "The kidneys, uterus, and bladder, were in a sound state.

7. "The thoracic viscera were also perfectly sound."

In the course of our remarks on the various modes of practice, exhibited under different titles of cases, treatises, &c. we have often had occasion to regret the backwardness of our cotemporaries in the use of the lancet. It is not for us to doubt that every thing which could be done for the benefit of the patient, whose case is above related, was in this instance tried; and if we *had* such doubts, we would suppress them rather than discourage such valuable and candid communications. But when pain and sickness are intense, our practice would always lead to immediate and copious bleeding; nor have we ever found reason to repent it. We are the more ready to make this remark, because that remedy was tried, and the attendant physicians seem doubtful whether they had not better have omitted it.

Article 5.—*Case of successful Amputation of the Uterus.* By JOSEPH CLARKE, M. D. Dublin.

THIS paper is not less valuable, on account of the candour of the writer, who informs us that the uterus having prolapsed was mistaken for a polypus, and as such, partly removed by ligature, and afterwards by the knife, when the disease was ascertained. The complete success of the operation will, we trust, induce surgeons to have recourse to it in such cases of cancerous uterus as are well ascertained, and admit of no remedy but the forlorn refuge of the most distressed of human beings.

Article 6.—*Case of Chorea Sancti Viti, cured by Purgatives.* Communicated by GEORGE KELLY, M. D.

Article 6.*—*Letter on the Application of Galvanism in the Cure of Congenital Deafness.* By ALEXANDER VOLTA, Professor of Natural Philosophy in the University of Pavia.

WHEN we consider how much we are indebted to Professor Volta for his improvement in this important novelty in physiology, we cannot cannot but respect every thing that comes from his pen on this subject. But the very slight and uncertain success of the only case here related, scarcely entitled the paper to a transcript. It is much to be regretted, that we have heard no accounts of any attempts in England to ascertain the effects of so powerful a remedy on subjects so numerous and so truly deserving of pity.

Article 7.—*Explanation of a supposed Case of Small-pox after Vaccination.* By HENRY JOHNSON, Fellow of the Royal College of Surgeons, Edinburgh.

THESE cases have occurred so often, that to relate them can only be useful in those neighbourhoods where an imperfect knowledge of the events has injured this valuable discovery. We shall, therefore only transcribe the author's reflections.

"Before concluding, I would beg leave to observe, that the practice of vaccine inoculation is beginning to be held by far too light; any body is supposed capable of performing an operation so trifling. Every surgeon knows how frequently he produces an inflammation of several days standing with a pustular appearance, without supposing he gives constitutional infection, and security against small-pox. I fear such a simple affection of the arm, is too often considered as the real and necessary infection. I am led to make this observation, from finding my own apprentices, when of very short standing indeed, frequently trying their skill in this way."

We perfectly agree with Mr. Johnson, that no one should undertake to vaccinate without a practical knowledge of the subject. But all the practical knowledge requisite appears to us a frequent sight of the vesicle in all its stages, with those few remarks from an experienced practitioner, which are easily retained by a learner of common attention. The various engravings which have at different times been offered to the public, have, in our opinion, done more harm than benefit. When the subject was new, such an illustration was proper and necessary for general information, and on account of the interest it excited. But, for practical purposes, nothing should be made use of but the best means of information.

Article 8.—*Case of Epilepsy cured by Trepanning.* By HENRY COOTES, of the Royal College of Surgeons in London, and Surgeon to the Salisbury Infirmary.

THIS should have been entitled "Case of Epilepsy, *occasioned by an injury to the cranium,* cured by trepanning *four years after the accident.*

The paper is valuable, but as all such cases are to be considered as Records, their utility is much increased by bringing forward the most remarkable circumstances attending them.

Article 9.—*Cases of Idiopathic Tetanus, with Observations.* By C. L. MURSINNA, Chief Surgeon to the Charité of Berlin.

IF this paper contained any thing particularly new, it would have been a sufficient apology for copying it from the Berlin "Newes

Journal

Journal fur die Chirurgie, &c." But we are not now to be taught that opium will sometimes cure locked jaw, especially when unconnected with a wound. Unfortunately also, we know how often it fails. Cases of this kind are well worth reading; but, when recorded, not of sufficient importance to find a place in two different Journals.

Article 10.—Contains some judicious Remarks "*On the Plan of Medical Reform.*"

The Enquirer adds his mite at the conclusion of the original articles. "On the Study of Mental Pathology."

The remarks on the difficulty with which this branch of medicine is attended are neither new, nor to be disputed any more than the imperfections of most of the plans hitherto pursued. But, when the writer assures us with confidence, that the principles of moral philosophy and metaphysics have been much advanced in modern times, we confess ourselves much at a loss to discover these advances. As to moral philosophy, we old-fashioned people are apt to be satisfied with the examples recorded of the Founder of our religion and his followers; and as to metaphysics, such is the Beotian dulness of most of us, that we are apt to rise from a grave discourse on these subjects, either without understanding our author, or, if we do understand him, with discovering that we have learned nothing but what was familiar to every thinking mind, before the writer dressed it in such a fashion, as to render it nearly obscure. We were somewhat gratified with the conclusion of the paper; not because it taught us any thing, but because it convinced us that the author, like ourselves, had every thing to learn.

"After these cursory remarks, it might have been proper, shortly, to state the advantages which are likely to accrue from examining the brain, and other organs, of those persons, who have been affected with mental disorders. But this field comprises so many important topics of discussion, that little benefit could be derived from any thing, which could be advanced here. Some of the principles, however, which should guide our researches into this subject, may probably be inferred from several of the preceding speculations.

"I have thus attempted to give a rough outline of a great and important subject; a subject, which not only involves consequences peculiarly interesting to the practical physician, but to every man who is anxious to acquire a scientific knowledge of human nature."

We mean not to undervalue such disquisition, and are free to acknowledge, our own sheets are not always as full of information as we could wish. But, from the gravity of the Enquirer, we are apt to expect something more pointed. These are all the original articles. The Review is useful in containing analyses of several foreign works. We hope the whole of Professor Reil's proposals for "instructing a set of popular practitioners in the common routine routine of practice" will be translated. We recommend it to be circulated by the Reforming Society, as a means of adding something more to their Reports, than a mere tedious detail of the insufficiency of practitioners in various parts of the country. Every medical man is aware how desirable some reform would be; but if competition is the spirit of improvement, we must be careful not to lay the foundations of monopoly.

---

*The Vaccine Contest; or, "Mild Humanity, Reason, Religion, and Truth, against fierce, unfeeling Ferocity, overbearing Insolence, mortified Pride, false Faith, and Desperation;" being an exact Outline of the Arguments and interesting Facts, adduced by the principal Combatants on both Sides, respecting Cow-pox Inoculation, including a late official Report on this Subject, by the Medical Council of the Royal Jennerian Society: chiefly designed for the Use of Clergymen, Heads of Families, Guardians, Overseers of the Poor, and other unprofessional Readers, who may be concerned for the Welfare of Mankind.* By William Blair, M. A. Surgeon of the Lock Hospital and Asylum, the Bloomsbury Dispensary, and New Rupture Society, Member of the Royal College of Surgeons, and of the Medical Societies of London, Paris, Brussels, Aberdeen, &c. &c. &c. 8vo. Murray, London.

The words in this long title page, which are included within commas, are taken from Dr. Rowley's publication, against which the present performance is chiefly directed. The manner in which this has been undertaken, and the motives which gave rise to it, are explained in the following paragraph, extracted from the preface.

" A mere glance at this book awakened attention; and a deliberate perusal of it, suggested the idea of turning against an implacable adversary the murderous weapons which he himself had provided for a different purpose. The author judged it would not be lost time, though a nauseous and revolting task, to extract the *marrow* or *quintessence* of that extraordinary performance; and, by placing the Doctor's own language in a new and vivid light, to afford a spirited and glowing picture of its genuine deformities. The real character and motives of an opponent, who is so entirely devoid of justice and decorum, cannot be better discovered, than by dissecting, analysing, and exposing to public view, what may be called the vitals and sinews, the internal springs, the peculiar features and tone of his composition. And, if it should be found, that his character and motives are far from pure, except in his own eyes, it may be questioned whether his pretended Truths be *unexceptionable*, or his *alledged* Facts such as honesty demands."

The work is formed into a dialogue between a country Curate, Dr. Bragwell, and a town Surgeon. The first applies to Saville Row for further particulars relative to the Doctor's objection against the cow-pox. This produces such a conversation as might be expected: though the feeble arguments of Dr. Bragwell only confirm confirm the clergyman in his good opinion of vaccination; yet, to bring forward parts of the subject unknown to one, and untouched by the other, the Surgeon is at this moment introduced as an interlocutor; we shall select a passage from this part of the work, to give our readers a specimen of the manner in which the whole is conducted.

Surgeon.—Reverend Sir, I greatly approve of your determination, and I bear witness this day to the ingenuousness and candour of your deportment towards Dr. Bragwell; of whom, and to whom, I am come at present to say a few words. I am a surgeon, residing in London, and well known to ——

Doctor.—Speak on, Sir; speak on; I am the sworn advocate of truth; and if I cannot convince the Reverend Gentleman, it is not for want of painstaking and zeal on my part. I am not ashamed of what I have said; and have told him nothing that has not been already published to all the world, in the third edition of my unanswerable work, or in my other practical writings. Not a sentence, Sir, nor a single word, do I retract. Come Sir, come, Sir, speak on. You, Mr. Surgeon, have you too joined these "*violent enthusiasts?*" You, of whom I had hoped better things? Why, Sir, you have been a strenuous defender of the truth, in former times: it was you, chiefly, who confronted and silenced the Philoacidi a few years ago; and I will presently either silence you, or chase you from my doors, as I advise all persons to do with incorrigible vaccinators. "I am happy to say, I never in one instance recommended vaccination;" and I have told this clergyman, that "the opposers of cow-pox are ready to meet its advocates, being secure of a glorious victory."

Surgeon.—Be assured, that if ever you *meet* them, it will be to your confusion and disgrace: but, such a meeting is needless; for, your heap of anti-vaccinarianism is before the public. You have raked together into one mass (a disgusting collection!) all the conjectures or hearsay tales, all the pretended cases of failure or disaster, which any person would communicate for the gratification of your vanity; with all the adverse facts hitherto published by others, though answered again and again. So that, whoever gives you, Dr. Bragwell, a proper reply, will have refuted the objections of every adversary that has appeared in this combat.

Doctor.—Well, well, Sir; go on with your invective. "I have no time for controversy." The Truth lies in a narrow compass. You have, of course, read my collection of Facts, and examined them attentively. Very true, Mr. Surgeon; in refuting me, you will vanquish the whole host of *Anti-vaccinarians.* Did you ever hear of the "Anti-vaccinarian Society?

Surgeon.—I am happy to have an opportunity of telling you, and this Reverend Gentleman, (whose patience equals his liberality of sentiment,) that your "Matters of Fact," and the "Anti-vaccinarian Society," of which you are known to be the *fac totum*, disgrace the medical profession; and impeach the moral ral characters of men of all ranks in the Faculty, who are far, very far your superior in reputation and virtue. Your publication is a *gross libel* (I repeat it) upon professional gentlemen of the first name and celebrity, and merits no other epithet than a most scandalous vehicle of falsehoods, tending to the worst consequences in society. You pique yourself on being the leader and father of the "Anti-vaccinarian Society:" and it is no wonder you should foster this darling of your own begetting. *Doctor Squirrell* (alias *Mr. S. the Apothecary*) is worthy of being your colleague in this establishment; which, indeed, looks too much like a mercenary contrivance, to bring the dissatisfied patients to your shop! It is perfectly of a piece with your self-commendatory puffs in the daily newspapers; your multiform circular hand-bills; your numerous placards on the dead-walls of the town; your acknowledged "public exhibitions in the lecture-room; your cart-loads of children in Saville-row;" your insidious bribes, feastings, and humiliating attentions to apothecaries, &c. who have been artfully seduced to countenance your plans!

PARENT.—Oh! does the learned Oxonian stoop so low as all this? Can he, then, be respected among his medical associates? But, I don't understand what kind of an institution Doctor Bragwell's "*Anti-vaccinarian Society*" is, of which he makes his boast.

SURGEON. *(aside.)*—Reverend Sir, you seem not to know any thing of the estimation in which this Physician is held, among really honourable men in London. I say nothing about his underlings, or the self-dubbed Doctors who have enlisted into his service. You may conjecture what kind of an establishment his "Anti-vaccinarian Society" is, by an advertisement I shall now read for your information."

As nothing has hitherto been written in this form, we have no doubt that Mr. Blair's endeavours will be useful in forwarding the important objects of this valuable discovery.

---

*Remarks on Mr. Birch's "Serious Reasons for uniformly objecting to the Practice of Vaccination."* By JAMES MOORE, Member of the Royal College of Surgeons in London. 8vo. Murray, 1807.

*An Answer to Mr. Birch, containing a Defence of Vaccination.* By JOHN RING, Member of the Royal College of Surgeons in London, and of the Medical Societies of London and Paris. 8vo. Murray.

HAVING already noticed Mr. Birch, our Readers may, perhaps, be surprised that his performance should have provoked two answers. They are, however, both of them characteristic of the writers. The first is gentlemanly, and contains as much novelty as the subject will admit: the second partakes of the usual warmth and asperity of the writer, for which, it must be confessed, the number of weak points in Mr. Birch afford ample scope.

